# Development of Nutrition Literacy Scale for Middle School Students in Chongqing, China: A Cross-Sectional Study

**DOI:** 10.3389/fnut.2022.888137

**Published:** 2022-05-20

**Authors:** Tiankun Wang, Mao Zeng, Changxiao Xie, Yuzhao Zhu, Zumin Shi, Manoj Sharma, Yong Zhao

**Affiliations:** ^1^College of Public Health and Management, Chongqing Medical University, Chongqing, China; ^2^Research Center for Medicine and Social Development, Chongqing Medical University, Chongqing, China; ^3^Department of Human Nutrition, College of Health Sciences, QU Health, Qatar University, Doha, Qatar; ^4^Department of Environmental and Occupational Health, School of Public Health, University of Nevada, Las Vegas, NV, United States; ^5^Chongqing Key Laboratory of Child Nutrition and Health, Children's Hospital of Chongqing Medical University, Chongqing, China

**Keywords:** assessment tools, Chongqing, Delphi, middle school students, nutrition literacy

## Abstract

**Background:**

Information on nutrition literacy of middle schoolers is limited and tools for measuring nutrition literacy of middle schoolers are inadequate. Nutrition literacy has a positive effect on health. Improving children's nutrition literacy can help them to master the necessary nutritional knowledge, develop a healthy lifestyle, and learn to supplement nutrition according to their own needs for healthy growth.

**Objectives:**

To develop the Chongqing Middle school student Nutrition Literacy Scale (CM-NLS).

**Methods:**

Three experiments were conducted. A theoretical framework and an initial item pool of CM-NLS were established based on the literature review. And the two-round Delphi method was used to explore the suitable acceptance indicators and items. Item evaluation and reduction were performed using the classical test theory. Then, the items in the final CM-NLS were tested for their validity and reliability amongst 462 middle school students. The construct validity was assessed using exploratory factor analysis (EFA) and confirmatory factor analysis (CFA). The internal consistency reliability and split-half reliability were evaluated using Cronbach's alpha coefficients.

**Results:**

The final CM-NLS consisting of 52 items that were based on three primary items (functional, interactive and critical) and six sub-items (obtain, understand, apply, interact, medial literacy and critical skill) was developed and validated. EFA suggested six factors explaining 69.44% of the total variance (Kaiser–Meyer–Olkin test = 0.916, Bartlett's test χ2 = 5,854.037, *P* < 0.001). CFA showed that the model fit the data adequately, with χ2/df = 1.911, root mean square error of approximation = 0.063, goodness-of-fit index = 0.822 and adjusted goodness of fit index = 0.790. The total CM-NLS Cronbach's alpha values of internal consistency and split-half reliability were 0.849 and 0.521, respectively, with reasonable reliability.

**Conclusions:**

CM-NLS is a valid and reliable instrument for assessing nutrition literacy among middle school students in Chongqing. Specifically, it could be used by practitioners for needs assessment before the implementation of a nutrition education program.

## Introduction

The prevalence of childhood obesity has been on the rise in recent years (1). The Global Burden of Disease data showed that approximately 107.7 million children worldwide were obese in 2015 (1) and nearly 124 million children are expected to be obese by 2025 in the world (2), 49.48 million by 2030 in China (3). According to studies, childhood obesity is closely associated with chronic non-communicable diseases, such as hypertension, diabetes and cardiovascular disease (4–6). The important factors affecting childhood obesity, such as children's dietary patterns (number, regularity, duration of meals and combination of food groups), ultra-processed foods (formulations of food substances often modified by chemical processes and then assembled into ready-to-consume hyper-palatable food and drink products using flavors, colors, emulsifiers and other cosmetic additives) (7) consumption habits and food choices, are strongly related to nutrition literacy (8–10).

Nutrition literacy is an emerging term defined as the comprehensive ability to obtain, understand and apply nutritional information (11, 12). Nutbeam classified the comprehensive ability into three levels from low to high: Functional nutrition literacy (FNL), interactive nutrition literacy (INL), and critical nutrition literacy (CNL). The definition of FNL emphasizes basic literacy and numeracy skills, INL refers to the ability to recognize and communicate nutritional information, and CNL refers to the ability to critically analyze nutritional information (13). Children with high levels of nutrition literacy could develop healthy dietary habits and food purchasing behavior (e.g., eat more vegetables and less cookies or candy) (14), whereas low nutrition literacy has been shown to be associated with unhealthy diets (e.g., purchase and consumption of high-calorie food) (9, 15, 16).

Although nutrition literacy has emerged as an area of increasing research focus in many countries, only a limited number of tools for assessing children and adolescents are available. The existing nutrition literacy assessment tools mainly focus on the evaluation of adults (17–20). In 2017, Asakura et al. developed the Nutrition Knowledge Questionnaire for primary school children consisting of a four-part test based on the understanding of terms, awareness of dietary recommendations, using the information to make dietary choices, awareness of diet-disease associations (21). The Adolescent Nutrition Literacy Scale developed by Bari contains 29 attitude statements under three sub-dimensions (22). Food and Nutrition Literacy evaluated the cognitive and skill domain, which was used to measure the validity and reliability of food and nutrition literacy amongst Iranian children (17). But these tools lack cognitive assessment and critical analysis of nutritional information. In China, Liao LL et al. compiled an evaluation tool suitable for college students according to Dietary Guidelines in Taiwan (23). The Peking University team has established nutrition literacy core items for preschool children (24, 25). However, culture and social environment can shape attitudes and beliefs and therefore influence nutrition literacy (26). And different from most cities, the foods in Chongqing are mainly spicy, which leads to a different focus of the questionnaire. Thus, the national questionnaire could not be fully applied for regional survey (27).

This study aimed to develop the scale to assess the nutrition literacy of middle school students and confirm it's validity and reliability, and provide references for evaluating the nutrition literacy of middle school students in Chongqing, China. It could be used by practitioners for needs assessment before the implementation of a nutrition education program.

## Methods

### Study Design and Participants

**Figure 1:**
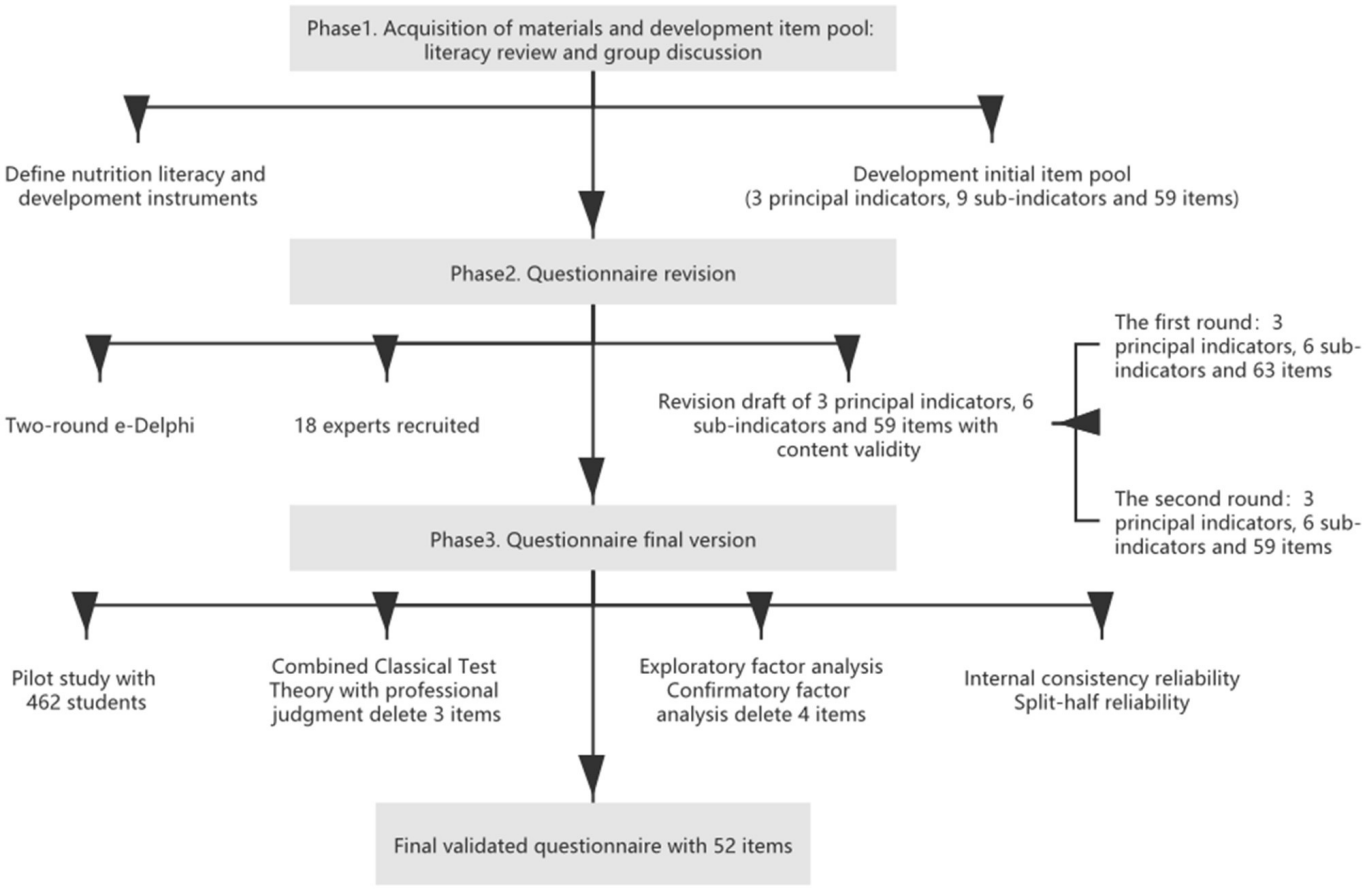
Flow chart of the questionnaire development procedure.

The study consisted of three consecutive phases. In the first phase, the Chongqing Middle school student Nutrition Literacy Scale (CM-NLS) test pool items were developed. In the second phase, 18 experts working in nutrition education-related fields were invited for a two-round e-Delphi to complete the construction of the framework and initial items. In the third phase, 462 middle school students (the age range of middle schoolers is generally between 12 and 18 years old in China) (28) were tested in the pilot study, the items were revised and the reliability and validity of CM-NLS were tested. Figure 1 shows the process of questionnaire development.

#### Phase 1: Literature Review and Design of Initial Items

##### Literature Review

A comprehensive electronic database search was conducted in PubMed, Web of Science and China National Knowledge Infrastructure from inception to January 2020 to retrieve relevant articles published in English and Chinese. “Nutrition literacy” and “Nutritional literacy” were used as search keywords to collect articles. A total of 242 articles were retrieved from CNKI, 186 articles were retrieved from Web of Science and 74 articles were retrieved from PubMed. In terms of article types, original articles and reviews on nutrition literacy were included. In terms of article content, all studies on the development of nutrition literacy assessment tools were included in the reading, focusing on the definition, fields and dimensions of nutrition literacy, and finally 43 articles were included. In summary, this step was used to guide the conceptual framework construction and initial item generation.

##### Design of Initial Items

The framework was designed based on Nutbeam's hierarchical model of health literacy, with classical functional, interactive and critical levels (29). The content was designed based on the definition of nutrition literacy (the ability of individuals to obtain, understand and process/apply basic nutrition information) (30–32) and other dimensions of existing nutrition literacy assessment instruments (23, 33–37). Functional nutrition literacy includes two acquisition skills, 18 understanding skills and 24 application skills. Interactive nutrition literacy includes one interactive skill, two emotional skills and four discussion skills. Critical nutrition literacy includes four items of media literacy, three items of analytical skills and three items of evaluation skills (Supplementary Table 1).

#### Phase 2: Assessment of Content Validity by Two Rounds of Expert Consultation

The purpose of this step was to identify if the questionnaire items generated in the previous step were sufficiently acceptable, comprehensive, and relevant to nutrition literacy from the perspective of professionals. The instrument was designed with Chinese, a total of 28 academics and professors proficient in Chinese were invited to evaluate the concepts of nutrition literacy by E-mail. To make it more specified and reliable, we made some inclusion criteria for experts enrolled in our study, including: with bachelor or higher degree; at least 5 years working experience in children's nutrition education; willing to take part in this study. According to the inclusion criteria, 18 experts (mean age = 41.67 years, mean years of working = 12.28 years) were finally included to meet the sample size requirements. They were asked to comment on the importance, feasibility of each item using a 5-Likert scale (1 = Not at all important, 2 = Low importance, 3 = Neutral, 4 = Moderately important, 5 = Extremely important). Importance means that the index is very important to measure the nutrition literacy of middle school students and must be included in the index system. Feasibility refers to whether the index is feasible to measure the nutrition literacy of middle school students.

Both Rounds of Delphi consulting were conducted by the same group of experts. After the first round of expert consultation, the framework of secondary items was adjusted according to the opinions: 1) The original secondary items “2.1 interactive skills, 2.2 emotional skills, 2.3 discussion skills” were merged into “2.1 interactive skills”; 2) Merge the original secondary items “3.2 analytical skills, 3.3 evaluation skills” into “3.2 critical skills”. After the second round of expert consultation, we deleted, added and modified the items according to the experts' opinions (delete four items: “Understand the channels for obtaining nutritional information” “easily obtain nutrition-related knowledge and skills” “no significant difference in nutrient content between white eggs, red eggs, native eggs and foreign eggs” and “the degree of trust in nutritional information on the media”. The critical skills of “understanding the definition of healthy weight and correct understanding of one's body shape” in critical nutrition literacy should be adjusted to the application skills in functional nutrition literacy), a pool of 59 items was formed to measure three domains (functional, interactive and critical) and six components (obtain, understand, apply, interact, medial literacy and critical) of nutrition literacy (Supplementary Table 2).

#### Phase 3: Pilot Study and Confirmation of Validation

After the two-round Delphi method was conducted, the CM-NLS, which includes 21 single-choice questions, three multiple-choice questions, one order question and 34 Likert-type questions, was used in 462 middle school students to confirm the validity of the scale. Each question in the scale has a corresponding score, the scores were divided into low and high levels based on their median scores, and higher total score represents higher nutrition literacy of students.

##### Data Collection

The convenience sampling method was used to recruit students from a middle school in Chongqing from June 2020 to July 2020. The research design of this study utilized a uniform resource locator invitation, with a Quick Response code embedded, providing access to the questionnaire to participate. The students who were asked to fill out the questionnaire, anonymous, without mentioning compensation. Literature demonstrated that the nutrition literacy level of middle school students in China, was ~15.6% (38). According to the formula of sample size calculation:


(1) $$\begin{array}{r} N=\left({Z_{\alpha }}^{2}\times p\times q\right)/d^{2}\; \end{array}$$

We set *p* = 0.156, *q* = 1—*p* = 0.844, and margin of error *d* = 0.30 × *p* = 0.2532, Z_α_ = 1.96; the calculated sample size was 231, considering the possibility of a 10% non-response rate, the minimum sample size that was needed for this study was calculated to be 254. In the survey, the actual total sample size in the survey included 462 individuals.

Based on the inclusion criteria (middle-schoolers who are able to read and understand the questionnaire; agree to participate in the questionnaire survey) and exclusion criteria (children with endocrine disorders diagnosed by doctors; children with central nervous system injury; children with secondary obesity caused by other reasons; unable or refuse to fill in the questionnaire), we excluded those who were unwilling to participate and did not fill in the questionnaires completely. Most participants completed this scale for approximately 15 min.

##### Item Selection

The techniques of Classical Test Theory (CTT) were applied to measure some observable information (scale scores) to obtain insights into variables (nutrition literacy level of middle school students) that could not be directly observed (39). The following eight criteria were used to determine the questions included in the tool (40):

1) Frequency analysis: In the responses of all questions, the selection of any option accounts for more than 80% should be deleted.

2) Coefficient of variation: The Coefficient of variation of items ≥0.25 or standard deviation >0.9 should be deleted.

3) Discriminant validity: According to the scores of 27% percentile before and after the total score of the scale (41, 42), the high and low groups were divided and the items with no difference in scores between the two groups were deleted (*P* > 0.05).

4) Intra-class correlation coefficient: The correlation coefficient (R) between all items under the same sub-indicator and the items with R value low than 0.2 or >0.9 were deleted.

5) Entry–dimension consistency: The R between all items under the same sub-indicator and the items with R value low than 0.2 or >0.9 were deleted.

6) Item-dimension: The R between one item and one dimension is less than the R between the item and other dimensions should be deleted.

7) Factor analysis: The maximum factor load <0.4 or absolute value of cross-loading <0.1 or each common factor item containing <3 items should be deleted.

8) Cronbach's alpha coefficient: If an item was deleted and a significant increase was present in the alpha coefficient, then deletion was considered.

Items with three or more of the above criteria for deletion should be deleted.

##### Reliability

The reliability of the scale was evaluated by internal consistency (Cronbach's alpha) and split-half reliability was used to represent the reliability of the total scale and each dimension.

##### Face Validity

The face validity of the questionnaire was verified in accordance with the evaluation of middle students and experts. At the end of the questionnaire, an open question was set, mainly by asking the respondents about the questions they encountered when filling out the questionnaire to know whether the items were measuring what we were intended to measure.

##### Content Validity

In this study, content validity index was used to evaluate the content validity. 18 experts were asked to measure the correlation between each item of the scale and the index content of the scale and were required to use 0 (no) and 1 (yes) to evaluate whether the items could properly reflect the nutrition literacy of students. The items with the content validity index value of 0.7 or above were kept (43) (Supplementary Table 2).

##### Construct Validity

Confirmatory factor analysis (CFA) and exploratory factor analysis (EFA) were used to evaluate the structural validity on Likert-type items. EFA is usually used to generate the factor structure between measured variables by using a group of samples, while CFA is used to extract another group of samples from the population to test the fit of the hypothetical factor structure. We randomly split the sample into two parts, with half of the sample size using EFA method to generate factor structures and the other half using CFA method for formal comparison of models (44). Principal component analysis was firstly performed unrotated by using maximum likelihood extraction and (determining the number of factors to retain) the number of factors was determined using the following criteria: the eigenvalue-greater-than-one rule (K1), the Kaiser–Meyer–Olkin statistical value is between 0.5 and 1, Bartlett's test results of correlation matrix *P* < 0.05 (45). Then, CFA was used to validate the content and characteristics of the basic constructs, whilst the choice of items was validated using EFA. Evidence of model fit was evaluated using indices of absolute fit and goodness-of-fit index. The reasonable threshold levels of these indices for CFA were considered as χ^2^ test results *P* > 0.05, χ^2^/df <3, root mean square error of approximation <0.08, goodness-of-fit index >0.9 and adjusted goodness of fit index >0.9 (44).

### Ethical Statement

This study adhered to ethical research standards and regulations. Ethical approval was obtained from the Ethics Committee of Chongqing Medical University. All participants were informed about the study and provide informed consent before participation in the Delphi expert consultation and pilot study.

### Statistical Analysis

We examined the Cronbach's alpha coefficient with SPSS 22.0 to evaluate internal consistency. CFA and EFA were conducted with maximum likelihood estimation using AMOS 23.0 to validate the structural validity on Likert-type items, whilst the tetrachoric correlation of STATA was used for binary items. Model fit was determined by comparative fit index and root mean square error of approximation, the overall goodness-of-fit of the model was used as the evaluation criteria of validity. Frequencies and means were utilized to describe the participant demographic characteristics and nutrition literacy. The percentage correct and standard deviation for each item were also calculated. SPSS 22.0 and STATA 16.0 were used to perform all data analyses.

## Results

### Participants

A total of 462 students participated in the pilot study (response rate = 99.14%). Their age (mean ± SD) was 13.38 ± 1.05 years, amongst which 218 (47.2%) were males and 244 (52.8%) were females. The average time of filling out the questionnaire was 14.71 ± 2.32 min. Amongst the participants, 197 (42.6%) were in boarding schools and 368 (79.7%) were non-singleton children. Their parents' education level was mainly junior high school. The most common occupational category was professional technician (Table 1).

**Table 1:** Basic demographic characteristics.

**variables**	***N***	**%**
Age (mean, SD)		13.38	1.05
Gender	Male	218	47.2
	Female	244	52.8
Ethnic	Han	454	98.3
	Others	8	1.7
grade	7	192	41.6
	8	270	58.4
Whether a boarding	Yes	197	42.6
	No	265	57.4
Whether an only child	Yes	94	20.3
	No	368	79.7
Number of children in the family*	2	223	61.0
	≥3	145	39.0
Primary caregiver	Father	290	62.8
	Mother	397	85.9
	Grandparents	65	14.1
	Relatives	25	5.4
	Others	13	2.8
Father's education level	Primary schools and below	55	11.9
	Junior high school	238	51.5
	Senior high school/technical secondary school/vocational high school	94	20.3
	College/bachelor degree or above	51	11.0
	Don't know	24	5.2
Mother's education level	Primary schools and below	91	19.7
	Junior high school	222	48.1
	Senior high school/technical secondary school/vocational high school	77	16.7
	College/bachelor degree or above	47	10.2
	Don't know	25	5.4
Father's career	Party and government officials	19	4.1
	Enterprise personnel	85	18.4
	Professional and technical personnel	181	39.2
	Agricultural laborer	32	6.9
	No employment or layoff	46	10.0
	Don't know	99	21.4
Mother career	Party and government officials	7	1.5
	Enterprise personnel	63	13.6
	Professional and technical personnel	176	38.1
	Agricultural laborer	70	15.2
	No employment or layoff	42	9.1
	Don't know	104	22.5

* *Refers to a student who is not an only child in the house*.

### Item Analysis Based on CTT

Item analysis was conducted by CTT, and the instrument included 14 items that did not meet the criteria. However, after the theoretical relevancy of nutrition literacy and the results of group discussion were combined, the final scale included 56 items (three items removed: Q7_1.2.2, Q8_1.2.3 and Q12_1.2.7). Examples of changes made based on item analysis are shown in Supplementary Table 3.

### Reliability

The Cronbach's alpha coefficient of the scale was tested for internal consistency. The results showed that the Cronbach's alpha coefficient of the total scale was 0.849, whilst that of the six subscales was 0.648–0.942, indicating that the reliability of internal consistency of the scale was stable. The total scale split-half reliability coefficient was 0.521, whilst that of the six subscales was 0.509–0.914 (Table 2).

**Table 2:** Cronbach's α coefficient for the CM-NLS scale.

	**Number of items**	**Scores**	**Mean**	**SD**	**Cronbach's α**	**Split-half reliability**
**1. Functional NL**	**35**	**0–99**	**69.70**	**11.17**	**0.826**	**0.503**
1.1 Obtain	3	0–12	8.21	2.48	0.819	0.765
1.2 Understand	14	0–32	21.67	4.62	0.648	0.509
1.3 Apply/Use	18	0–55	39.81	7.10	0.778	0.724
**2. Interactive NL**	**5**	**0–20**	**5.46**	**3.75**	**0.942**	**0.914**
2.1 Interact	5	0–20	5.46	3.75	0.942	0.914
**3. Critical NL**	**12**	**0–48**	**27.07**	**10.39**	**0.938**	**0.860**
3.1 Medial literacy	8	0–32	17.37	7.85	0.938	0.901
3.2 Critical	4	0–16	9.71	3.57	0.909	0.906

### Validity

#### Content Validity

The mean value of content validity index containing 56 items was 0.91, indicating good content validity. Group discussion, expert consultation, and the face validity results of the pilot study proved that most of the items in the questionnaire were suitable for middle school students.

#### Construct Validity

##### CFA

**Figure 2:**
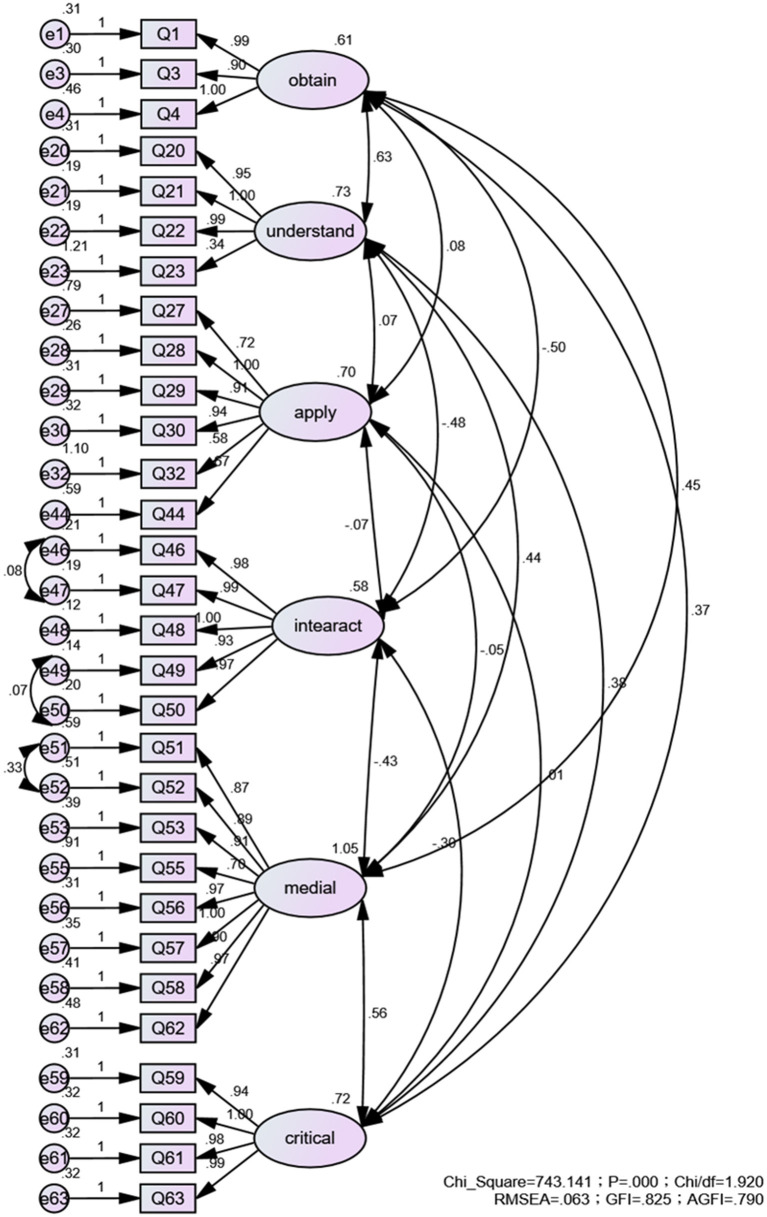
First-order CFA analysis factor loading construct validity study for CM-NL.

**Figure 3:**
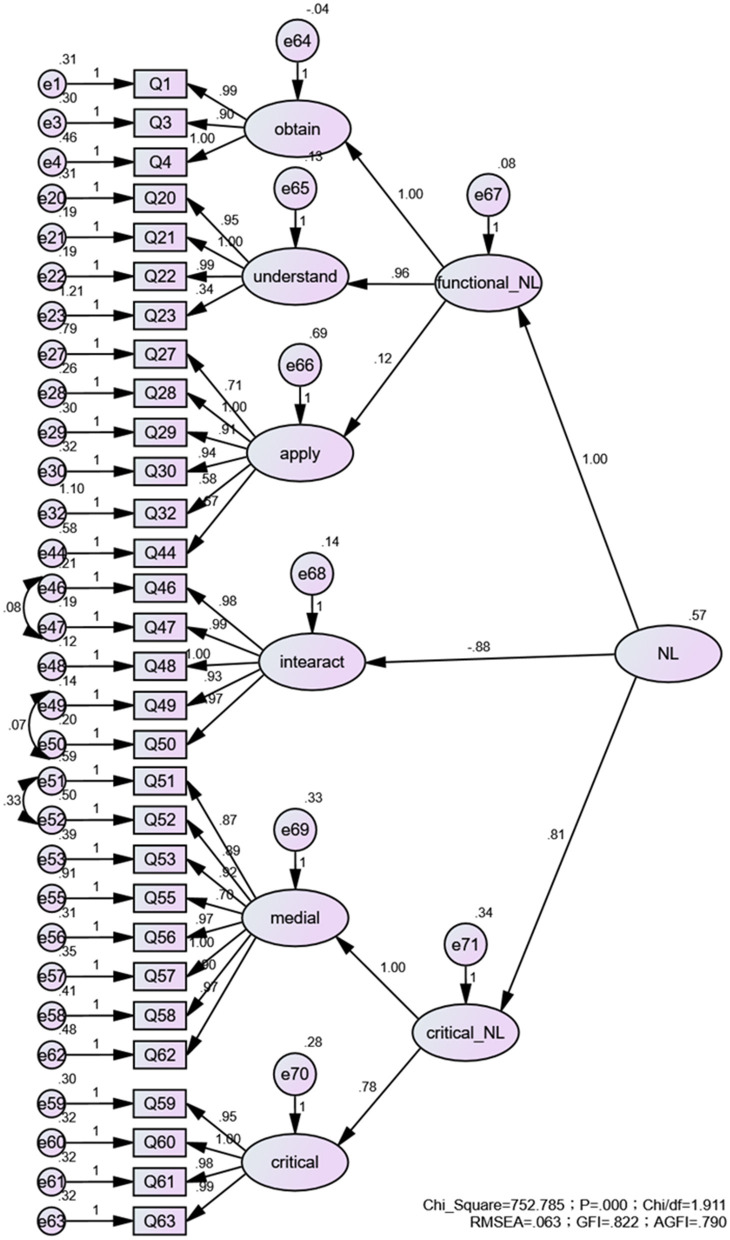
Second-order CFA analysis factor loading construct validity study for CM-NL.

Significant correlations were found between items and factors in the first-order model (*P* < 0.001). In the second-order model, except that the factor loading between application skills and FNL was not statistically significant (*P* = 0.123), all the others were statistically significant (*P* < 0.001). The results of the model fit for the CFA of scale are reported in Figures 2, 3, which indicated a desirable fit of the proposed models.

##### EFA

The KMO test showed sampling adequacy = 0.916 and Bartlett's test confirmed that EFA was appropriate (χ2 = 5854.037, *P* < 0.001). The percentage of the total variance was 69.438%, with six rotated factors. Based on the results of EFA and professional knowledge, seven specific sub-items were modified and adjusted: (1) the following four application skill (Q31_1.3.8, Q33_1.3.10, Q34_1.3.11, Q41_1.3.18) items were deleted. (2) Three items from “interactive skills” and “critical skills” were changed to “medial literacy (ML)” (Q51_2.1.6, Q52_2.1.7, Q62_3.2.4). The finalized CM-NLS consisting of 52 items that were based on three primary items (functional, interactive and critical) and six sub-items (obtain, understand, apply, interact, medial literacy and critical skill).

## Discussion

This study was the first to report the validity and reliability of the assessment instrument to comprehensively evaluate the level of nutrition literacy of middle school students in Chongqing. Based on the conceptual models of health literacy illustrated by Nutbeam, nutrition literacy was divided into FNL, INL and CNL (28). After a two-round Delphi survey, the items of the nutrition literacy scale of middle schoolers in Chongqing were finally determined, including three primary items, six sub-indicators and 52 items. The results of the research confirmed the reliability and validity of CM-NLS, including content and structure.

The experts consulted in Delphi had a high degree of agreement on the FNL. The experts consulted in Delphi had a high degree of agreement on the highest score for FNL. Other researchers also believed that functional literacy is the basis of interactive and critical literacy (31). FNL is important for middle schoolers to acquire nutrition knowledge and develop healthy dietary habits. Besides, middle schoolers should obtain and understand nutrition information, such as understanding the whole process of food from farm to table (production, processing, transportation, purchase and handling). A notable detail that the authors agreed with Thomas, who revealed that nutrition literacy should also emphasize the importance of practice in nutritional information (30, 46). However, the practical ability of middle school students in China was insufficient, they were relatively weak in nutritional practical skills (47, 48). This may be due to the fact that in most cases, the caregiver in China may be the only one who purchases and prepares food. They think children should focus on the study of theoretical knowledge in middle school and achieve good results in exams, resulting in less opportunities for middle schoolers to purchase and cook food. Therefore, the “application skills” mainly emphasized dietary behavior whilst ignoring the importance of students' application of nutrition information. This problem should be given attention in the future. In addition, the INL score was high. According to the results of the pilot study, middle school students mostly obtained information from families and teachers; it was a one-sided communication between teachers and parents to students, leading to the lack of initiative of students to interact with others. Although the CNL score was the lowest amongst the three subscales, the students' media literacy and critical skills are still necessary to understand. In recent years, adolescents have increasing access to nutrition information (49), which may lead to information misunderstanding and confusion (50). Wadsworth advocated that media literacy (the ability to critically view and understand information) should be included in diet-related education (51). In the framework concept, media literacy and critical skills were placed in the section of CNL, which made up for the deficiency that only a few researchers had explored (17).

After CTT and structural analysis, some items differing from the results of expert consultation and the purpose of the research were deleted or adjusted. For instance, the item “It is easy to understand the contents of the Dietary Guidelines for Chinese Residents” was deleted. This deletion could imply that the Dietary Guidelines for Chinese Residents was difficult to understand for middle school students with lacking professional guidance to understand (52). The CFA results showed that except for the *P*-value, goodness-of-fit index and adjusted goodness of fit index were close to 0.9, whilst other data met the requirements. This finding may be influenced by the sample size; the chi-square value of the model was very sensitive to the sample size and the larger the sample size was, the more likely it was to reach a significant value (53, 54). The EFA and CFA results showed a poor correlation between application skills and the other five dimensions. The application skills should not belong to FNL and be contrary to the judgment of professional knowledge. This finding could be interpreted as three factors at interplay in obtaining the results. Firstly, according to Bloom's taxonomy (revised), the six levels of cognitive learning are remembering, understanding, applying, analyzing, evaluating and creating (55). The cognitive development of middle school students may not be advanced enough to “apply” the skills (56). Secondly, the application skills may have some regional differences in the real world; the original literature and guidelines focused on adolescents across China but Chongqing is a multi-ethnic area (57). Finally, the Chinese education system places more emphasis on route learning and silent learning rather than application, leading to the original hypothesis not being verified (58). The Cronbach's alpha value of the screened scale was 0.851; the values of the other items were between 0.648 and 0.942 and the mean value of content validity index containing 56 items was 0.91, indicating that CM-NLS was consistent with the theoretical framework and had reasonable reliability and validity.

This study is based on literature research. A total of 18 experts were consulted in strict accordance with the Delphi method. The results combined subjective assessment with objective analysis. Furthermore, CTT and validity tests were used to screen the items and the relationship amongst theory, experience and data in scale design was well-handled (40). However, the study still has several limitations. Firstly, the sample size was not representative of the entire population. As CM-NLS only included students from one school, it still needs to be applied to a larger population to verify its feasibility and validity. Besides, due to the COVID-19 epidemic situation, the preliminary survey of this study was mainly conducted online, and some information bias may be present in the self-report of students. Furthermore, we did not set the question about if the students received a class about nutrition or foods, which would make it difficult to carry out measures to improve their nutrition literacy in the future, and the design of nutrition literacy assessment instruments is dynamic and needs to be modified and improved under the updated guidelines and literature.

Overall, CM-NLS is a valid and reliable instrument to measure nutrition literacy amongst middle school students in Chongqing. It could be used to identify key problems, such as nutrition education intervention, and pertinence measures to education. It could also provide a scientific basis for the implementation of nutrition education strategies. We will further use effective and widely used nutrition literacy questionnaire to verify the applicability and effectiveness of the nutrition literacy assessment tool in the future.

## Data Availability Statement

The raw data supporting the conclusions of this article will be made available by the authors, without undue reservation.

## Ethics Statement

The studies involving human participants were reviewed and approved by the Ethics Committee of Chongqing Medical University. Written informed consent to participate in this study was provided by the participants' legal guardian/next of kin.

## Author Contributions

TW, MZ, and YZha jointly conceptualized the study. MZ and YZha were involved in data extraction and verification of the extracted data. TW and MZ worked on the first draft of the manuscript and amended the second draft of the manuscript. CX, YZhu, and MS provided guidance and suggested revisions. YZha provided critical updates to the final manuscript. All authors read and approved the final manuscript.

## Funding

This work was supported by the Chinese Nutrition Society (Science Popularization and Communication Research Fund project, Award Number CNS-SCP2020-34).

## Conflict of Interest

The authors declare that the research was conducted in the absence of any commercial or financial relationships that could be construed as a potential conflict of interest.

## Publisher's Note

All claims expressed in this article are solely those of the authors and do not necessarily represent those of their affiliated organizations, or those of the publisher, the editors and the reviewers. Any product that may be evaluated in this article, or claim that may be made by its manufacturer, is not guaranteed or endorsed by the publisher.
