# Supplementary material for: Development of Nutrition Literacy Scale for Middle School Students in Chongqing, China: A Cross-Sectional Study
Source: Front Nutr. 2022 May 20;9:888137. doi: 10.3389/fnut.2022.888137 (PMC9165640; doi:10.3389/fnut.2022.888137)
Supplement: Supplementary file 1 [file Data_Sheet_1.docx]

Supplementary Material

**Table 1. Indicators of nutrition literacy and their connotations**

| **Domain/Dimensions components** | **Domain/Dimensions define** | **The first round** | | **The second round** | |
| --- | --- | --- | --- | --- | --- |
|  |  | Materiality | Feasibility | Materiality | Feasibility |
| **1.** **Functional NL** | **Capacity to access, understand and use nutritional information.** | **4.59±0.51** | **4.29±0.85** | **4.97±0.07** | **4.74±0.38** |
| 1.1 Obtain | Ability to seek, find and obtain nutritional information. | 4.56±0.71 | 4.50±0.86 | 4.85±0.29 | 4.70±0.41 |
| 1.2 Understand | Ability to have basic knowledge of nutrition and understand general nutritional information. | 4.61±0.61 | 4.56±0.78 | 4.86±0.34 | 4.71±0.41 |
| 1.3 Apply/Use | Ability to apply nutritional information to daily life to achieve a healthy diet. | 4.82±0.40 | 4.29±0.92 | 4.68±1.01 | 4.24±1.02 |
| **2. Interactive NL** | **Capacity to take action to obtain and exchange nutrition information that improves health through interaction and engaging in various forms of communication.** | **4.65±0.49** | **4.12±0.86** | **4.73±0.39** | **4.50±0.46** |
| 2.1 Interact | Ability to interact with nutritional information from others (peers, family members, or nutritionists) to promote healthy dietary habits. | 4.11±0.76 | 4.06±1.00 | 4.73±0.39 | 4.43±0.49 |
| **3.** **Critical NL** | **Capacity to critically evaluate and appraise nutritional information, advice and recommendations from various sources with the right perspective.** | **4.38±0.81** | **4.19±1.05** | **4.83±0.30** | **4.32±0.60** |
| 3.1 Medial literacy | Ability to critically interpret and use nutritional information in the media. | 4.24±0.90 | 3.82±1.02 | 4.69±0.40 | 4.08±0.59 |
| 3.2 Critical | Ability to critically analyze and use nutritional information in specific situations. | 4.59±0.80 | 3.89±0.90 | 4.76±0.40 | 4.21±0.56 |

**Table2. Initial items on Chongqing Middle school student nutrition literacy scale (CM-NLS)**

|  |  |  | **CCT†** | | | | | | | | |
| --- | --- | --- | --- | --- | --- | --- | --- | --- | --- | --- | --- |
|  | **Scale items** /**Question** | **CVI** | **1** | **2a** | **2b** | **3** | **4** | **5** | **6** | **7** | **8** |
|  | **1.** **Functional NL** |  |  |  |  |  |  |  |  |  |  |
|  | *1.1 Obtain* |  |  |  |  |  |  |  |  |  |  |
| Q1_1.1.1 | I will seek answers when I don't know anything about nutrition. **[liket-5]** | 0.88 |  |  |  |  |  |  |  |  |  |
| Q2_1.1.2 | I know where to get nutritional information. **[liket-5]** | — | — | — | — | — | — | — | — | — | — |
| Q3_1.1.3 | Know where to find accurate information when I have a nutrition-related problem or want to learn healthy dietary behaviors. **[liket-5]** | 1.00 |  |  |  |  |  |  |  |  |  |
| Q4_1.1.4 | It is not difficult for me to find the needed nutritional information from a large number of information sources. **[liket-5]** | 1.00 |  |  |  |  |  |  |  |  | **X** |
| Q5_1.1.5 | I can easily acquire nutrition-related knowledge and skills. **[liket-5]** | — | — | — | — | — | — | — | — | — | — |
|  | *1.2 Understand* |  |  |  |  |  |  |  |  |  |  |
| Q6_1.2.1 | Learn about food sources and categories **[sorting problem]** | 0.94 |  |  |  |  |  |  |  | X | X |
| Q7_1.2.2 | Rich in calcium and easily absorbed by the body, the first food is **milk and dairy products**. **[single-select]** | 0.81 |  |  | X | X | X | X |  | X | X |
| Q8_1.2.3 | A soft drink is not milk. **T** | 0.88 |  |  | X | X | X | X |  | X | X |
| Q9_1.2.4 | Whole grains (millet, corn, etc.) are more nutritious than refined grains (rice, flour, etc.). **T** | 0.88 |  |  | X |  |  | X |  |  |  |
| Q10_1.2.5 | There was no significant difference in nutrient content between white eggs, red eggs, native eggs, and foreign eggs. **T** a | — | — | — | — | — | — | — | — | — | — |
| Q11_1.2.6 | know the advantage that eats bean curd, soya-bean milk to wait for soybean product**[multi-select] ﹡** | 1.00 |  |  | X | X |  |  |  | X |  |
| Q12_1.2.7 | Iodization of salt can prevent goiter. **T** | 0.88 |  |  | X |  | X |  |  | X |  |
| Q13_1.2.8 | Smoking and salting foods can increase the risk of cancer. **T** | 0.88 |  |  | X |  |  | X |  |  |  |
| Q14_1.2.9 | Being overweight or underweight increases the risk of disease. **T** | 0.81 | X |  | X |  |  |  |  |  |  |
| Q15_1.2.10 | When eating meat, try to eat lean meat, fat meat can eat, but cannot eat more. **T﹡** | 0.94 | X |  | X |  |  | X |  | X |  |
| Q16_1.2.11 | Good dietary habits can prevent chronic diseases such as hypertension and diabetes. **T** | 0.94 | X |  | X |  |  |  |  |  |  |
| Q17_1.2.12 | You can reduce your exercise if you eat less. **F﹡** | 0.88 | X |  | X |  | X | X | X |  | X |
| Q18_1.2.13 | Drink water in small quantities more than once. **T﹡** | 0.94 | X |  | X |  |  | X |  |  |  |
| Q19_1.2.14 | Separate meals can help prevent mouth-to-mouth diseases. **T﹡** | 0.94 | X |  | X |  | X |  |  |  |  |
| Q20_1.2.15 | It is easy to understand the contents of the Dietary Guidelines for Chinese residents. **[liket-5]** | 0.94 |  |  |  |  |  |  |  |  | X |
| Q21_1.2.16 | It is easy to understand the nutritional information (such as energy, protein, sugar, etc.) on food packaging. **[liket-5]** | 0.81 |  |  |  | X |  |  | X |  |  |
| Q22_1.2.17 | It is easy to understand that recommendations relating to health and nutrition in secondary school students. **[liket-5]** | 0.88 |  |  |  | X |  |  | X |  |  |
| Q23_1.2.18 | It is easy to understand nutrition information you read in a brochure, book, or on the Internet. **[liket-5]** | 0.88 |  |  |  | X |  |  | X |  |  |
|  | *1.3 Apply/Use* |  |  |  |  |  |  |  |  |  |  |
| Q24_1.3.1 | When eating, consider **nutrition** first. **[single-select]** | 1.00 |  |  | X |  |  |  |  | X |  |
| Q25_1.3.2 | Use nutrition labels to choose foods wisely. **[multi-select]** | 0.94 |  |  |  |  |  |  |  | X |  |
| Q26_1.3.3 | Choose snacks wisely. **[multi-select]** | 0.88 |  |  |  | X |  |  |  | X |  |
| Q27_1.3.4 | Don't substitute fruits for vegetables. **[liket-5]** | 0.94 |  |  |  |  |  |  |  |  |  |
| Q28_1.3.5 | Don't replace fresh vegetables with pickles and pickles. **[liket-5]** | 0.94 |  |  |  | X |  |  |  |  |  |
| Q29_1.3.6 | Don't snack instead of meals. **[liket-5]** | 0.88 |  |  |  | X |  |  |  |  |  |
| Q30_1.3.7 | Do not use canned fruit, preserved fruit, and other processed fruit products instead of fresh fruit. **[liket-5]** | 0.94 |  |  |  | X |  |  |  |  |  |
| Q31_1.3.8 | Frozen foods should not be thawed at room temperature. **[liket-5]** | 0.81 |  |  |  |  |  | X |  |  | X |
| Q32_1.3.9 | Cooked food should be kept at room temperature for no more than 2 hours. **[liket-5]** | 0.81 |  |  |  |  |  |  |  |  |  |
| Q33_1.3.10 | Keep raw and cooked food separately. **[liket-5]** | 0.88 |  |  |  |  |  |  |  |  | X |
| Q34_1.3.11 | Food should be heated through the second time. **[liket-5]** | 0.88 |  |  |  |  |  |  |  |  | X |
| Q35_1.3.12 | Eat a variety of foods: eat at least 12 foods a day and 25 foods a week. **[single-select] ﹡** | 0.94 |  |  |  | X |  | X |  |  | X |
| Q36_1.3.13 | Eat breakfast every day. **[single-select]** | 0.94 |  |  | X |  |  |  |  | X |  |
| Q37_1.3.14 | Eat fruit every day. **[single-select]** | 0.94 |  |  |  |  |  |  |  |  |  |
| Q38_1.3.15 | Drink milk every day. **[single-select]** | 0.94 |  |  |  |  |  |  |  |  |  |
| Q39_1.3.16 | Avoid or limit sugary drinks. **[single-select]** | 0.94 |  |  | X |  |  |  |  |  |  |
| Q40_1.3.17 | Don't drink. **[single-select]** | 0.88 |  |  | X |  |  |  |  | X |  |
| Q41_1.3.18 | Regular meals. **[liket-5]** | 1.00 |  |  |  | X |  | X |  |  |  |
| Q42_1.3.19 | Cut down on Western fast food. **[single-select]** | 0.94 |  | X | X |  |  |  |  |  |  |
| Q43_1.3.20 | Cut down on fat, smoke, and spicy foods. **[single-select] ﹡** | 0.94 |  | X | X |  |  | X |  |  |  |
| Q44_1.3.21 | Do not patronize food and drink stalls such as roadside stands. **[liket-5]** | 0.94 |  |  |  | X |  |  |  |  |  |
| Q45_1.3.22 | Knowledge of healthy weight and can rightly realize body shape. **[single-select] ﹡** | 0.81 |  |  | X |  |  | X | X | X | X |
|  | **2. Interactive NL** |  |  |  |  |  |  |  |  |  |  |
|  | *2.1 Interact* |  |  |  |  |  |  |  |  |  |  |
| Q46_2.1.1 | An active attitude toward obtaining nutritional information. **[liket-5] ﹡** | 0.81 |  |  | X | X |  |  | X |  |  |
| Q47_2.1.2 | Communicate willingness to learn about nutrition and health. **[liket-5] ﹡** | 0.94 |  |  | X | X |  |  | X |  |  |
| Q48_2.1.3 | Willingness to receive nutrition education. **[liket-5]** | 0.88 |  |  | X |  |  |  |  |  |  |
| Q49_2.1.4 | Willingness to change poor dietary habits using the nutrition knowledge learned. **[liket-5]** | 0.81 |  |  | X | X |  |  |  |  |  |
| Q50_2.1.5 | The willingness to persuade others to change their bad dietary habits. **[liket-5]** | 1.00 |  |  | X |  |  |  |  |  |  |
| Q51_2.1.6 | Talk about nutrition with others (e.g. friends, family, etc.). **[liket-5]﹡** | 0.94 |  |  |  |  |  | X | X |  | X |
| Q52_2.1.7 | Take the initiative to disseminate nutrition knowledge to others. **[liket-5]** | 0.88 |  |  |  |  |  |  | X |  | X |
|  | **3.** **Critical NL** |  |  |  |  |  |  |  |  |  |  |
|  | *3.1 Medial literacy (ML)* |  |  |  |  |  |  |  |  |  |  |
| Q53_3.1.1 | Attention to nutritional information in the media. **[liket-5]** | 0.94 |  |  |  |  |  |  |  |  |  |
| Q54_3.1.2 | Believe that the nutrition information reported by the media (e.g., school health education courses, Internet, books/brochures, television) is correct. **[liket-5]** | — | — | — | — | — | — | — | — | — | — |
| Q55_3.1.3 | Critical of nutrition information from all sources in society. **[liket-5]** | 0.75 |  |  |  |  |  |  |  |  | X |
| Q56_3.1.4 | It can judge the accuracy and scientific nature of nutrition-related information in the media. **[liket-5]** | 0.94 |  |  |  |  |  |  |  |  |  |
| Q57_3.1.5 | When confronted with contradictory nutritional information, one can judge whether the information is right or wrong through media reports. **[liket-5]** | 0.94 |  |  |  |  |  |  |  |  |  |
| Q58_3.1.6 | The extent to which nutritional information in the media affects you. **[liket-5]** | 0.88 |  |  |  | X |  |  |  |  |  |
|  | *3.2 Critical* |  |  |  |  |  |  |  |  |  |  |
| Q59_3.2.1 | How easy it is to tell whether nutritional information is scientific or not. **[liket-5]** | 0.94 |  |  |  |  |  |  |  |  |  |
| Q60_3.2.2 | How easy it is to distinguish between healthy and less healthy foods. **[liket-5]** | 0.81 |  |  |  |  |  |  |  |  |  |
| Q61_3.2.3 | How easy it is to assess the impact of eating habits on health. **[liket-5]** | 0.81 |  |  |  |  |  |  |  |  |  |
| Q62_3.2.4 | Dare to question deeply rooted social and cultural phenomena related to food and health. **[liket-5]** | 0.94 |  |  |  |  |  |  | X |  | X |
| Q63_3.2.5 | When I am advised on healthy eating, I can judge what fits my health needs. **[liket-5]** | 1.00 |  |  |  |  |  |  |  |  |  |

21 single-choice questions, three multiple-choice questions, one order question and 34 Likert-type questions were included after two rounds of an expert consultation.

—representative was not included in the preliminary survey.

Bold indicates correct choices or scoring criteria.

X means to delete an entry based on judgment.

CVI: content validity index

†CTT: Classical Test Theory. 1 Frequency analysis; 2 Coefficient of variation (CV);3 Discriminant Validity; 4 Intra-class correlation coefficient (R); 5 Entry–dimension consistency; 6 Item-dimension R; 7 Factor analysis; 8 Cronbach’s alpha coefficient.

﹡According to CCT standards should be deleted, but combined with professional knowledge to retain the item (11 items).

The grey represents the deleted items (11 items).

**Table3. Each factor Rotated Component Matrix**

| **Item** | **factors** | | | | | |
| --- | --- | --- | --- | --- | --- | --- |
|  | **1** | **2** | **3** | **4** | **5** | **6** |
| Q51_2.1.6 | **0.847** |  |  |  |  |  |
| Q52_2.1.7 | **0.796** |  |  |  |  |  |
| Q57_3.1.5 | **0.787** |  |  |  |  |  |
| Q62_3.2.4 | **0.740** |  |  |  |  |  |
| Q56_3.1.4 | **0.739** |  |  |  |  |  |
| Q53_3.1.1 | **0.737** |  |  |  |  |  |
| Q58_3.1.6 | **0.687** |  | 0.305 |  |  |  |
| Q41_1.3.18 | **0.629** |  |  |  |  |  |
| Q55_3.1.3 | **0.623** |  |  |  |  |  |
| Q22_1.2.17 |  | **0.797** |  |  |  |  |
| Q23_1.2.18 |  | **0.782** |  |  |  |  |
| Q3_1.1.3 |  | **0.772** |  |  |  |  |
| Q21_1.2.16 |  | **0.760** |  |  |  |  |
| Q1_1.1.1 |  | **0.708** |  |  |  |  |
| Q4_1.1.4 |  | **0.667** |  |  |  |  |
| Q20_1.2.15 |  | 0.374 |  |  |  |  |
| Q49_2.1.4 |  | -0.322 | **-0.798** |  |  |  |
| Q48_2.1.3 |  | -0.373 | **-0.789** |  |  |  |
| Q50_2.1.5 |  |  | **-0.782** |  |  |  |
| Q47_2.1.2 | -0.335 | -0.304 | **-0.773** |  |  |  |
| Q46_2.1.1 |  | -0.337 | **-0.747** |  |  |  |
| Q29_1.3.6 |  |  |  | **0.896** |  |  |
| Q30_1.3.7 |  |  |  | **0.826** |  |  |
| Q28_1.3.5 |  |  |  | **0.800** |  |  |
| Q27_1.3.4 |  |  |  | **0.747** |  |  |
| Q44_1.3.21 |  |  |  | **0.607** |  |  |
| Q32_1.3.9 |  |  |  | **0.525** |  | -0.483 |
| Q61_3.2.3 | 0.305 |  |  |  | **0.815** |  |
| Q63_3.2.5 | 0.356 |  |  |  | **0.799** |  |
| Q60_3.2.2 |  |  |  |  | **0.778** |  |
| Q59_3.2.1 | 0.347 | 0.300 |  |  | **0.720** |  |
| Q34_1.3.11 |  |  |  |  |  | 0.732 |
| Q31_1.3.8 |  |  |  | 0.314 |  | -0.628 |
| Q33_1.3.10 |  |  |  |  |  | 0.623 |

Note: The gray mark represents the item that has changed
